# MOFs-Modified Electrochemical Sensors and the Application in the Detection of Opioids

**DOI:** 10.3390/bios13020284

**Published:** 2023-02-16

**Authors:** Jiaqi Zhao, Ying Kan, Zhi Chen, Hongmei Li, Weifei Zhang

**Affiliations:** 1 College of Materials and Chemistry, China Jiliang University, Hangzhou 310018, China; 2 Key Laboratory of Chemical Metrology and Applications on Nutrition and Health for State Market Regulation, Division of Chemical Metrology and Analytical Science, National Institute of Metrology, Beijing 100029, China

**Keywords:** MOFs, MOFs Composites, electrochemical sensors, microfluidic chips, opioids

## Abstract

Opioids are widely used in clinical practice, but drug overdoses can lead to many adverse reactions, and even endanger life. Therefore, it is essential to implement real-time measurement of drug concentrations to adjust the dosage given during treatment, keeping drug levels within therapeutic levels. Metal-Organic frameworks (MOFs) and their composite materials modified bare electrode electrochemical sensors have the advantages of fast production, low cost, high sensitivity, and low detection limit in the detection of opioids. In this review, MOFs and MOFs composites, electrochemical sensors modified with MOFs for the detection of opioids, as well as the application of microfluidic chips in combination with electrochemical methods are all reviewed, and the potential for the development of microfluidic chips electrochemical methods with MOFs surface modifications for the detection of opioids is also prospected. We hope that this review will provide contributions to the study of electrochemical sensors modified with MOFs for the detection of opioids.

## 1. Introduction

Chronic pain is a major public problem, with it being estimated more than 1 in 5 adults in America experienced chronic pain, and with a higher prevalence associated with advancing age [1,2]. Patients of various ages and vocations may experience substantial effects from pain on their physical, mental, and quality of life. Opioids are powerful analgesics that have been used for many years in clinical settings to control and decrease pain.

Opioids are substances, either naturally occurring or synthetically produced, that bind to certain receptors in the brain and body and dampen the strength of pain signals. It has been used as a class of drugs for moderate or severe pain, coughs, and diarrhea for hundreds of years. Additionally, because opioids can lessen short-term pain, they are also successfully utilized for pain relief in patients with active cancer, end-of-life care, and other pain relief treatments [3,4]. Opioids that are used often in clinical settings include morphine, fentanyl, oxycodone, buprenorphine, methadone, codeine, and others. Even though opioids are frequently used in clinical practice, real-time opioid concentration monitoring is crucial for clinical counseling about the safe use of opioids because opioids can result in drug addiction, physical and psychological dependence, and even death.

Opioid detection techniques include fluorescence [5], colorimetry [6], chromatography [7], surface-enhanced Raman spectroscopy [8], mass spectrometry [9], and liquid chromatography-tandem mass spectrometry [10]. Despite the fact that these techniques have shown good selectivity and accuracy in the detection of opioids, they have several potential limitations, such as complicated pre-treatment, specialist personnel, time-consuming, and expensive tests. The electrochemical detection method [11,12] has the advantages of being inexpensive, simple to use, sensitive and accurate, and quick. Furthermore, these sensors can also be integrated into microfluidic chips and employed as dependable portable or tiny devices for detecting requirements which can better match the needs of portable and rapid detection of opioids in judicial judgment and clinical application. However, issues like peak potential overlap, a high detection limit, and a constrained linear range may arise when using the unaltered bare electrode [13,14]. By modifying the bare electrode with materials that have high performance and safeguard the environment, the aforementioned issues can be resolved. Electrochemical sensors for opioid detection are now built using a variety of new materials, including metal and metal oxide nanoparticles, carbon nanotubes (MWCNTs), graphene, and MOFs [15]. MOFs have a wide variety of applications in the production of electronic equipment [16], sensors [17], energy storage [18], gas separation [19], supercapacitors [20], medication delivery [21], environmental purification [22], and other disciplines due to their high porosity, large surface area, and other benefits.

**Figure 1:**
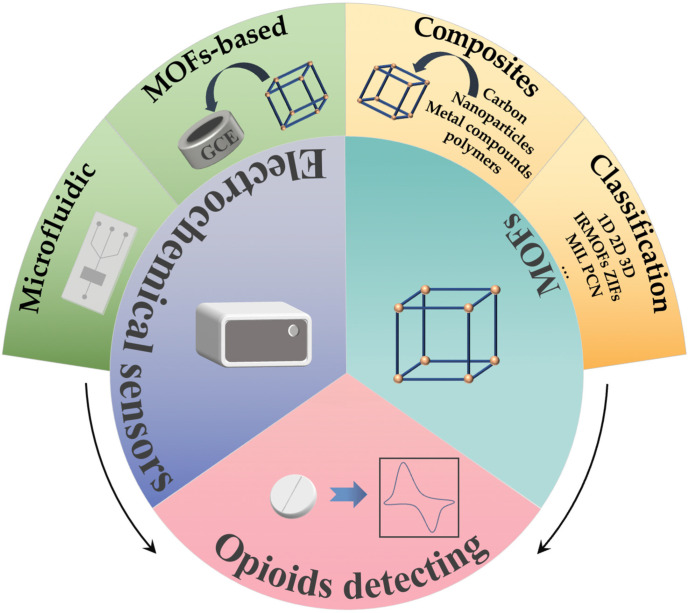
Schematic overview of the review.

Electrochemical sensors with modified MOF electrodes for the detection of opioids have been demonstrated with a low detection line, a broad linear range, and excellent selectivity. Due to the high integration achieved by microfluidic chips and electrochemistry, as well as their low sample and reagent consumption, microfluidic-based electrochemical sensors have been rapidly developed and applied in recent years, which enable the construction of small devices for rapid detection. Based on these findings, this review introduced the application of MOFs and MOFs complexes, their surface modified electrochemical sensors for the detection of opioids, as well as the application of microfluidic chips in combination with electrochemical methods. Besides this, the potential for the development of microfluidic chips electrochemical methods with MOFs surface modifications for the detection of opioids was also prospected, as shown in Figure 1.

## 2. MOFs Materials

### 2.1. Classification of MOFs

Yaghi et al. synthesized the first MOFs in 1995 [23]. MOFs have progressively entered the spotlight as a new functional material, which has been widely explored and employed in a variety of fields. Currently, with the development of a large number of different kinds of MOFs, there is a need to rationally classify them. MOFs can be classified in a variety of ways, as illustrated in Table 1. Here, we mainly review the first two classification methods.

**Figure 2:**
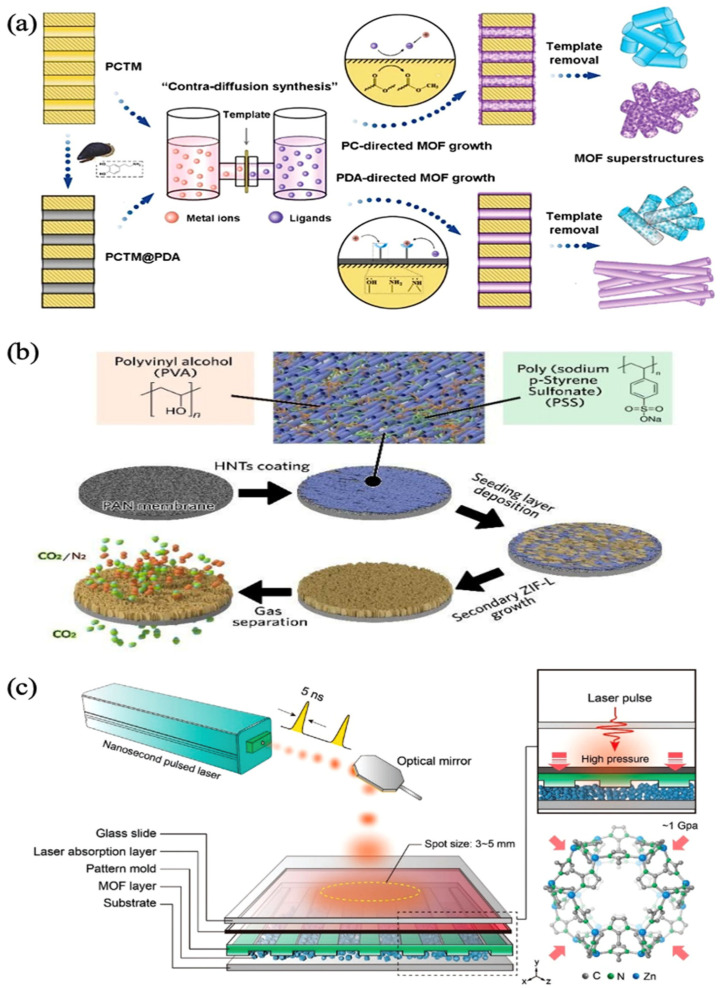
(**a**) Illustration of surface chemistry directed template synthesis of MOF superstructures using a contra diffusion method in pristine and PDA modified polycarbonate track-etched membrane (PCTM) templates. Reprinted from [25] with permission; (**b**) Schematic diagram of the HNTs membrane modification and ZIF-L vertical growth processes. Reprinted from [26] with permission; (**c**) The laser pulse is absorbed by the absorption layer and produces high pressure of up to 1 GPa in nanoseconds to print the patterns on the surface of MOF crystals. Reprinted from [27] with permission.

There is a strong connection between the properties of MOFs and the spatial composition of the skeleton. Chen et al. investigated the electrical conductivity of a group of nickel-based MOFs with the same metal ligands and found that the one-dimensional hybrid (1D) MOFs material had superior electrical conductivity [24]. Yu et al. obtained well-defined one-dimensional hybrid MOF nanotubes by using polydopamine (PDA) for the first time to regulate the reverse diffusion synthesis of the 1D MOF superstructure, as Figure 2a shows. A well-defined one-dimensional hybrid MOF nanotube was successfully obtained [25]. Li et al. utilized bottom-up template generation technique to manufacture continuous 2D MOF nanosheet coatings. These coatings have a vertical arrangement, which improves permeability and selectivity, like Figure 2b [26]. Liu et al. used a binder-free ultrafast laser to forge 3D MOF. The high strain rate of the laser enhanced MOF’s molding capabilities overcame brittleness and allowed the creation of arbitrary 3D nanostructures with a 100% higher mechanical strength than powdered nanoparticles. These structures can be used in separation, bio-medical applications, and motors, as in Figure 2c [27]. On the basis of the above, it is certain that the categorization of MOF structural properties by skeleton spatial structure is a clear classification approach that is advantageous to the use of MOFs in a variety of fields.

However, the skeleton space composition of the framework structure alone cannot provide information about core ions and organic ligands. The limitations of the classification methods are also expanded by the method based on ligands, which also clarifies the properties of various MOFs series. According to the different ligands, MOFs are divided into isoreticular metal-organic frameworks (IRMOFs), zeolitic imidazolate frameworks (ZIFs), materials of institut lavoisier (MIL), and porous coordination networks (PCN). Yaghi et al. used the extended network technique to successfully produce a new type of porous crystalline material known as IRMOFs-1 (also known as MOF-5), which involved coupling reticulated metal ions with organic hydroxylates. A metal-organic framework with similar network topology areIRMOFs The pore size and function of these materials can be altered according to the design system [28]. IRMOFs have been used in a diverse array of applications, including adsorption [29], catalysis, [30,31] drug delivery [32], and sensors [33], because of their unique qualities, such as a huge surface area and chemical stability. Park et al. synthesized ZIF-1 to ZIF-12 by co-polymerizing zinc or cobalt ions with imidazolate-type chains. The crystal structure of ZIFs is based on the non-jointed network structure of seven different aluminosilicate zeolites. Both ZIF-8 and ZIF-11 were subjected to in-depth research, and the results of this research showed that ZIF-8 and ZIF-11 have high thermal stability owing to the fact that their guests are released without breaking the skeleton. Furthermore, the results of gas adsorption research demonstrated unequivocally that ZIF-8 and ZIF-11 have a permanent void fraction [34]. They have a wide range of potential uses in a variety of scientific disciplines, including adsorption [35], catalysis [36], supercapacitors [37] and the treatment of diseases [38]. Additionally, Férey et al. synthesized MIL-100 by combining Cr^3+^ and trimeric acid under hydrothermal conditions. MIL was a porous metal phosphate prepared with trivalent cations of different elements, such as Fe^3+^, Al^3+^, Ga^3+^, In^3+^, V^3+^, Cr^3+^, and phosphate ligands. MIL-53’s framework is extremely flexible and may adopt various geometries depending on the intense host-guest interaction. These materials were characterized by high stability, large pores, uniform and permanent porosity, and large specific surface area [39,40]. In recent years, MIL has received increasing attention for applications in chemical substance adsorption [41], photocatalysis [42,43], sensors [44,45], etc. A porous coordination network (PCN) contained multiple cubic octahedral nanopore cages, and its application in storage devices has been intensively investigated due to its properties such as stable pore structure and adjustable pore size [46,47].

In addition, according to the different types of organic ligand, it can be divided into nitrogen-containing heterocyclic [48], carboxyl-containing [49], and nitrogen-containing heterocyclic and carboxyl-containing [50]. Considering the different compositions of materials, they can be divided into metal-organic framework (MOF-n), rare-earth polymeric framework (RPF-n) [51], and metal peptide framework (MPF-n) [52].

### 2.2. MOFs Composites

On the basis of MOFs, various materials may be loaded into and doped into to create new materials with variable compositions and sizes. These new materials are referred to as MOFs composites. MOFs composites contain unique optical, electrical, magnetic, and catalytic capabilities in comparison to plain MOFs. These characteristics compensate for the shortcomings of single MOFs and make MOFs composites more desirable. The common binary composites mainly include MOFs-carbon, MOFs-metal nanoparticles, MOFs-metal complexes, and MOFs-polymers.

#### 2.2.1. MOFs-Carbon

One of the reasons why MOFs-carbon complexes are more common in MOFs complexes is that, since there is still room to improve the stability and conductivity of MOFs, compounding carbon materials with stable structure and strong conductivity complement pure MOFs and enhance the sensing capability of both materials. Carbon materials exist in various forms with different properties, and also show different advantages in combination with MOFs in detection.

**Figure 3:**
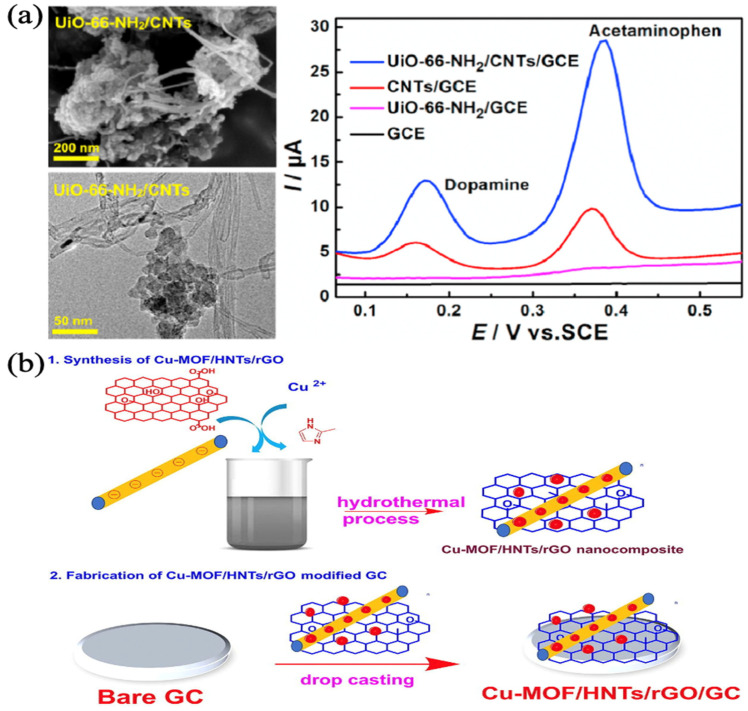
(**a**) SEM and TEM images of the UiO-66-NH2/CNTs nanocomposite, and DPVs of DA and AC at different electrodes. Reprinted from [53] with permission; (**b**) Synthesis and fabrication of Cu-MOF/HNTs/rGO nanocomposite. Reprinted from [54] with permission.

Li et al. synthesized UiO-66-NH_2_ by the hydrothermal method and prepared UiO-66-NH_2_/CNTs nanocomposites. CNTs and UiO-66-NH_2_/CNTs maintained tubular and octahedral structures, respectively, so UiO-66-NH_2_/CNTs composites have a large surface area, abundant active sites, and good electrical conductivity. The composites were modified on the glassy carbon electrode (GCE) surface to achieve simultaneous detection of dopamine (DA) and acetaminophen (AC) under the synergistic effect of UiO-66-NH_2_ and CNTs, and the results indicated detection limits of 15 nM and 9 nM, respectively, but the current peaks differed greatly when DA and AC were detected simultaneously, as in Figure 3a [53]. Manoj et al. prepared Cu-MOF-based nanocomposites by using a one-pot hydrothermal method. They compared the electrochemical properties of Cu-MOF/HNTs or Cu-MOF/HNTs/rGO nanocomposites modified on the GCE surface and found that the latter had a further reduced charge transfer resistance, which indicated that reduced graphene oxide (rGO) provided more conduction pathways in the composites, as in Figure 3b. The composites overcome the disadvantages of pure MOFs with poor electrical conductivity and particle aggregation. With the synergistic effect of active metal sites and high porosity of Cu-MOF, high conductivity of rGO, and large surface area of halloysite nanotubes (HNTs), the composite exhibited good electrochemical performance in the detection of DA and AC, with detection limits of 0.03 μM and 0.15 μM, respectively, and excellent separation of the anodic peaks of DA and AC [54].

#### 2.2.2. MOFs-Metal Nanoparticles

Common metal nanoparticles (MNPs), such as AuNPs, AgNPs, and PtNPs, are utilized extensively in the fields of catalysis, biomedicine, and environmental science, due to their high electrical conductivity and exceptional catalytic capabilities [55,56,57,58]. The development of MNPs, on the other hand, is primarily restricted to simple agglomeration. It is possible to successfully overcome the agglomeration problem of MNPs by using MOFs as carriers for encapsulating or loading MNPs. This can also improve the specific surface area of the composites, which in turn broadens the application fields of both materials.

**Figure 4:**
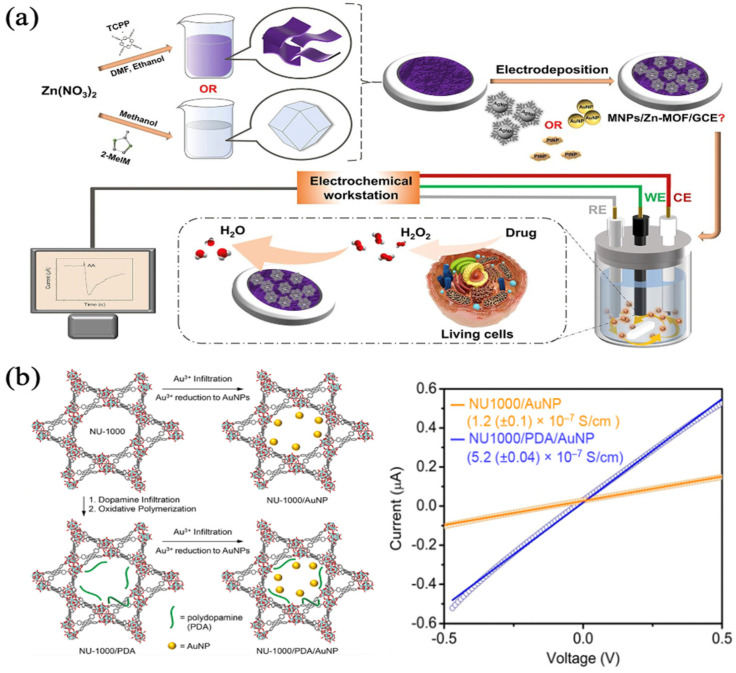
(**a**) Schematic diagram of the major steps for fabricating MNPs/Zn-MOF modified electrodes and the H_2_O_2_ sensor used for in situ monitor of living cells-released H_2_O_2_ from the stimulation of drug and transfer data to the electrochemical station. Reprinted from [59] with permission; (**b**) Transformation of NU-1000 to NU-1000/AuNP, NU-1000/PDA, and NU-1000/PDA/AuNP composites and representative I-V plots of NU-1000/AuNP (orange) and NU-1000/PDA/AuNP (blue). Reprinted from [60] with permission.

Chen et al. deposited AgNPs on 2D Zn-MOFs and 3D Zn-MOFs by electrodeposition and compared them. According to the findings, Ag/2D Zn-MOFs had a superior electrocatalytic activity compared to Ag/3D Zn-MOFs. Furthermore, the 2D MOFs increased the dispersion and stability of the active metal components, had a bigger specific surface area, and a higher conductivity, as in Figure 4a. When detecting H_2_O_2_, the linear range of the Ag/2D Zn-MOFs modified GCE was 5.0 μM–70 mM with a detection limit of 1.67 μM. More notably, the modified electrode could detect the response of live cells to H_2_O_2_ during specific drug stimulation in real time [59]. Gordillo et al. induced the reduction of percolated Au^3+^ ions to AuNPs by MOF and polymer, then transformed the porous but electrically insulating NU-1000 pure MOF material into NU-1000/AuNP and NU-1000/polydopamine/AuNP composites. Although the porosity of the composites was lower than that of the pure MOF material NU-1000, the large porosity was still maintained, whilst at the same time, the composites obtained significant electrical conductivity (~10^−7^ S/cm), which was 10^4^ times higher than the electrical conductivity of the MOF/MNPs composites under the same conditions so far, as in Figure 4b [60]. In the future, it may be used in the fields of electronics, electrocatalysis, and energy storage devices.

#### 2.2.3. MOFs-Metal Compounds

The numerous types of metal compounds each have their own unique properties, and the complexes that these metal compounds form with MOFs also have their own special features and are employed in a broad variety of diverse fields.

**Figure 5:**
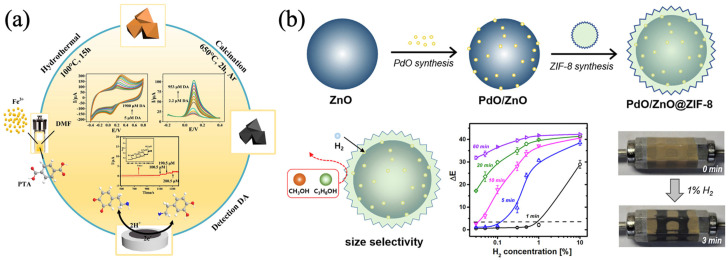
(**a**) Synthesis diagram of the Fe/Fe_3_O_4_@C and its electrochemical detection for DA. Reprinted from [61] with permission; (**b**) PdO/ZnO@ZIF-8 colorimetric sensor detect of hydrogen. Reprinted from [62] with permission.

Zhang et al. synthesized Fe/Fe_3_O_4_@C composites by using Fe-MOF as the precursor template and compared the sensing performance of the detection of DA by three electrochemical detection methods, cyclic voltammetry (CV), differential pulse voltammetry (DPV), and amperometric when the composites were modified on GCE. CV and DPV were superior to the amperometric method in terms of sensitivity and linear range. Furthermore, CV was more selective for DA in complex matrices such as in the presence of multiple sugars, urea, ascorbic, and Na^+^ and K^+^. Meanwhile, the sensor demonstrated a high level of stability as well as reproducibility, As in Figure 5a [61]. Hua et al. proposed a colorimetric sensor with ZIF-8 film encapsulated PdO/ZnO (PdO/ZnO@ZIF-8) to cope with the problem of naked-eye readable detection of hydrogen leakage. The yellow pigment of PdONPs could be oxidized by hydrogen to black Pd, which was an irreversible sensing material. The advantages of MOFs lead to superior performance in the selectivity, sensitivity, and detection speed of their encapsulation materials. Compared with bare PdO/ZnO, the composite material had a shorter response time (less than 1 min) to H_2_O_2_, with improved sensitivity and enhanced selectivity, as shown in Figure 5b [62].

#### 2.2.4. MOFs-Polymers

The combination of polymers and MOFs can achieve functional complementarity and compensate for the poor conductivity and stability of MOFs materials. Some applications of MOFs-polymers in the field of electrochemical detection are presented as an example of complexes composed of chitosan, Molecular Imprint Polymers (MIPs), and MOFs.

Chitosan, a polysaccharide containing N-acetyl-D-glucosamine and D-glucosamine units, is a polymer with the advantages of non-toxicity, high permeability, low cost, and stable covalent bonds [63,64]. Suk Choi et al. synthesized Co-hemin MOF from cobalt and heme, used porphyrin as an organic linker, coated Co-hemin MOF with chitosan by ultrasound, and surface modified GCE with Co-hemin MOF/chitosan by electrodeposition, thus the amino and hydroxyl groups in the chitosan structure could immobilize the enzyme with good application value. Thus, phanerochaete chrysosporium cellobiose dehydrogenase (CHD) was immobilized on the surface, and an electrochemical biosensor for lactose determination was established with a wide linear range of 10–100 mM and a detection limit of 4 mM [65].

**Figure 6:**
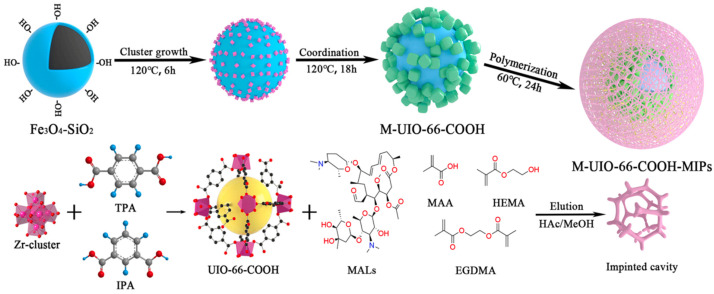
Synthetic diagram of M-UIO-66-COOH-MIPs. Reprinted from [68] with permission.

MIPs are polymers obtained by the polymerization of functional monomers. Specific recognition, high selectivity, high reproducibility and stability, and low cost are the significant advantages of their application in analysis and detection [66,67]. Liu et al. prepared Fe_3_O_4_ magnetic cores by a solvothermal method, and the activated M-UIO-66-COOH was prepared by a two-step coordination method and substrate selection, which was used as a substrate to explore the optimal synthesis conditions of MIPs composites. The MIPs were coated with magnetic metal-organic framework M-UIO-66@MIPs modified with hydroxyl groups, as in Figure 6, and the method was developed for the detection of five macrolide antibiotics (MALs) with detection limits ranging from 3.1–44.6 μg/L. Meanwhile, the selective and efficient adsorption of five MALs was achieved [68].

## 3. MOFs-Based Electrochemical Sensors

### 3.1. Construction of MOFs-Based Electrochemical Sensors

With the increased application of electrochemical analysis methods in clinical, food, and environmental monitoring, more and more research is devoted to improving the efficiency, linear range, detection limit, and selectivity of electrochemical detection. Detection with bare electrodes suffers from the problems of overlapping peak potentials, poor stability, and an insufficiently wide linear range. Surface modification of the original working electrode is a common means to improve the detection performance. Exploiting the various options of modifying electrodes, the shortcomings of the bare electrode can be compensated according to the demand, thus meeting different detection needs. MOFs are new materials, and their composites have been well developed. Constructing MOFs based and MOFs composite based surface modified electrodes for electrochemical sensors has numerous application and promising future prospects.

**Figure 7:**
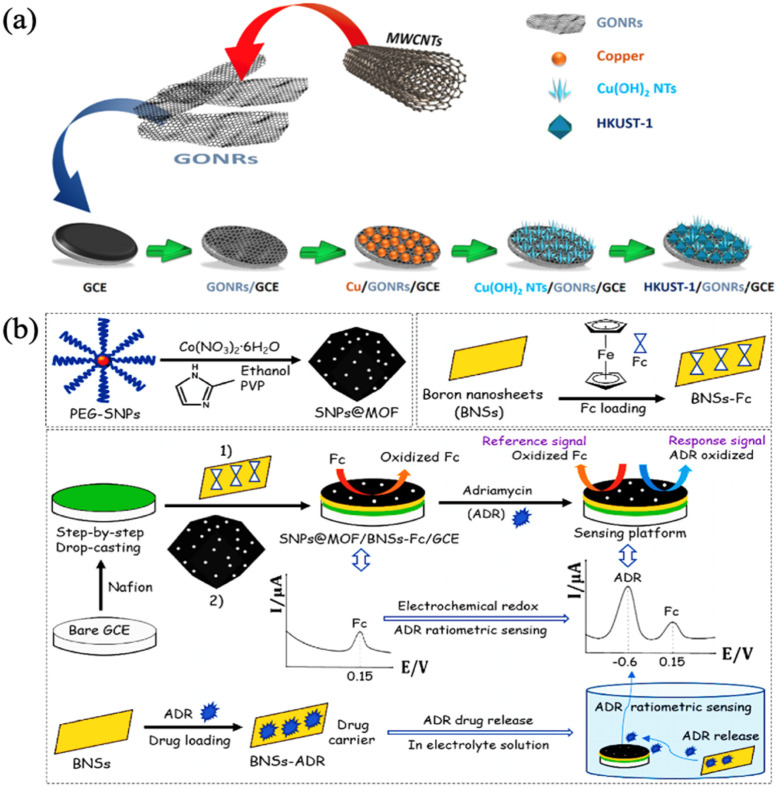
(**a**) Schematic illustration of the step-by-step fabrication of HKUST-1/GONRs/GCE. Reprinted from [70] with permission; (**b**) Schematic illustration for the preparation of a modified electrode platform SNPs@MOF/BNSs-Fc/GCE for ratiometric electrochemical sensing and real-time monitoring of drug release. Reprinted from [71] with permission.

Akhter et al. made a biosensor based on tri-metallic Co-Ni-Cu-MOF, which was grown on nickel foam (NF) using a conventional solvothermal method, and compared the performance to Co-MOF/NF, Ni-MOF/NF, and Cu-MOF/NF, respectively, used a determination of an anticancer therapeutic agent, nilutamide (NLM). Co-Ni-Cu-MOF/NF have shown a wide linear range between 0.5–70 μM and 70–900 μM and a low limit of detection of 0.48 ± 0.02 nM [69]. Jalal et al. used a simple chemical unzipping method to prepare graphene oxide nanoribbons (GONRs) with pristine MWCNTs and modified them on the surface of GCE, then grew HKUST-1 onto GONRs/GCE by a deposition method. Due to the synergic effect of the GONRs and HKUST-1 framework, HKUST-1/GONRs/GCE demonstrated superior performance in detection toward Imatinib (IMA) as an anticancer drug, which had two linear dynamic ranges, 0.04–1.0 μM and 1.0–80 μM, whilst the detection limit of IMA was 0.006 μM. In the determination of IMA in urine and serum samples, it was shown that it had a marvelous application prospect, as in Figure 7a [70]. Yang et al. synthesized sulfur nanoparticles (SNPs), and coated cobalt metal MOF, to obtain SNPs@MOF. At the same time, boron nanosheets (BNSs) were prepared by ultrasonic assisted liquid phase stripping and loaded with ferrocene (Fc) to form BNSs-Fc. The two materials were simultaneously cast on the surface of GCE to construct a dual-channel electrochemical sensor and real-time drug release monitoring platform, SNPs@MOF/BNSs-Fc/GCE. The detection of adriamycin (ADR) had stable Fc signal output and content-dependent ADR signal output. The detection limit of ARD was 2 nM, and the detection effective range was 0.01–10 μM. The testing of biological fluid samples proved the dependability, as in Figure 7b [71].

Since the properties of different drugs are different, it is necessary to explore the pH value of the optimal conditions for the detection process. The applicable pH for the detection of most opioids is 7, but since the oxidation peak potential of Morphine (MO) and Codeine (CO) tends to be negative around 7, becausethe oxidation mechanism is proton separation, and the sensitivity of the detection is highest at pH = 5. It is necessary to explore the most appropriate electrochemical detection conditions, which can improve the accuracy and performance of sensors in opioid detection to a certain extent.

### 3.2. Microfluidic Chips in MOFs-Based Electrochemical Sensors

In the meantime, electrochemical detection can also be built into a variety of portable and reliable micro-devices for a variety of applications, which will facilitate quicker detection. In recent decades, microfluidics has been widely utilized in assay technology and is acknowledged as one of the most promising analytical tools. It has many significant advantages, including component miniaturization, microliter level volume, ease of operation, low cost, and automation [72]. With the rapid development of microfluidics, it is now possible to design portable and robust detection devices by combining microfluidics with electrochemical analysis methods. These detection devices can satisfy a wide range of needs.

**Figure 8:**
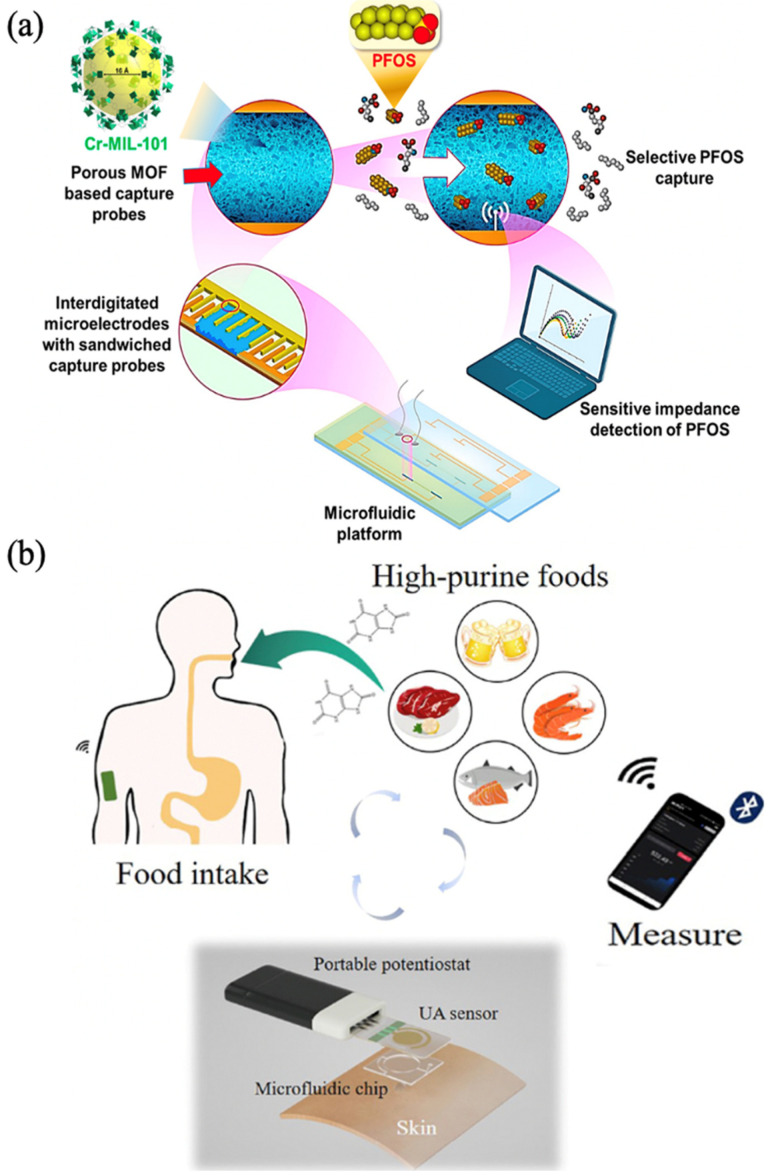
(**a**) Schematics of PFOS Detection. Reprinted from [73] with permission; (**b**) A complete wearable sensing system consists of a portable potentiostat, uricase@MAF-7-based sensor, and microfluidic chip for sweat sampling. Reprinted from [74] with permission.

In recent years, researchers have focused on integrating MOFs into microfluidics to create biosensors with the advantages of both MOFs and microfluidics. Microfluidic biosensors based on MOFs have a large surface area, large pore volume, and rich composition, and, when combined with electrochemical methods, y can achieve superior detection performance. Cheng et al. prepared Cr-MIL-101 composites by the hydrothermal method and interdigitated microelectrodes (IDμE) on standard glass slides of 25 mm × 75 mm × 1 mm. Finally, Cr-MIL-101 composites were packed in microchannels of microfluidic chips sandwiched between IDμE. An impedance electrochemical sensor in situ detection method based on the synergistic MOF and the microfluidic platform was constructed. Among them, the Cr-MIL-101 composite was used as a probe to capture perfluorooctanesulfonate (PFOS) based on targeting affinity, and the sensitivity of detection was improved by encapsulating the probe in a microfluidic platform. For in situ analysis of PFOS, this method had a detection limit of 0.5 ng/L, which was comparable to the state-of-the-art in situ technique, as in Figure 8a [73]. Xiao et al. prepared the metalazolate framework-7 (MAF-7) material and encapsulated the uricase in it. Uricase@MAF-7 was modified on the surface of activated gold electrodes. The uricase@MAF-7-based assay could detect uric acid (UA) levels ranging from 2 μM to 70 μM. The detection limit was as low as 0.34 μM. Then a chip with microchannel was prepared using PDMS, and a non-invasive wearable device was constructed by integrating a uricase@MAF-7-based electrochemical sensor. Microfluidic chips and wireless electronic readout devices were used to realize real-time, accurate, and sensitive detection of UA in human sweat during movement, as in Figure 8b [74].

## 4. Application of MOFs-Based Electrochemical Sensors for Opioid Concentration Detection

Although opioids are effective painkillers, their clinical use is somewhat limited because of the serious side effects they cause. These side effects include addiction, respiratory depression, and constipation. Additionally, a concentration of opioids in the blood that is higher than their effective level can lead to death due to respiratory depression. Electrochemical analysis is a relatively inexpensive method, and its detection speed is fast, allowing it to replace traditional detection methods, whilst the performance of bare electrodes for detection is also enhanced by MOFs used as surface modification materials, as Table 2 shows.

Because of their extremely high porosity, wide surface area, and high level of stability, MOFs are ideally suited for use as electrode modifiers for opioid medicines. Naghian et al. prepared Zn (II)-MOF material and completed the modification of the electrode by drop casting of Zn (II)-MOF suspension on the surface of the screen-printing electrode (SPCE). To determine the optimal conditions for the detection of Fentanyl, the effects of scan rate and pH on voltammetry were investigated to determine the best experimental conditions. The detection limit of fentanyl aqueous solution was 0.3 μM in the concentration range of 1–100 μM tested by the DPV method, and low doses of fentanyl were successfully determined in plasma and urine [76]. Moreover, because the modified electrode was SPCE, it could be used as a disposable sensor, which avoided the problem of cross-contamination.

Due to the diversity of MOFs composites, different MOFs composites are selected to obtain different performance modifiers, and a reasonable selection of MOFs composites can be made to meet different testing requirements. Yahyapou et al. synthesized Zn/La^3+^ MOFs with an average grain size between 30–80 nm by microwave co-precipitation, and prepared Zn/La^3+^/MOF/MIP/GCE by electropolymerization in combination with MIPs to detect buprenorphine (BUP). The optimal monomer concentration, BUP concentration, pH, number of cycles, and applied potential conditions were explored in the system with BUP as the template, pyrrole as the monomer, and potassium ferricyanide as the electrochemically active tracer to obtain the optimal response parameters. The response curves were finally obtained in the concentration range of 4–50 ng/mL in the linear range with the detection limit as low as 1.08 ng/mL, and the determination of the BUP concentration in the actual blood samples was achieved [75]. Akbari et al. prepared Hydroxyapatite (HA) Multi-Walled MWCNT and Cu-H_3_BTC materials and then prepared MWCNT-HA/Cu-H_3_BTC composite. The MWCNT-HA/Cu-H_3_BTC composite was characterized by X-ray diffraction, field emission scanning electron, Fourier transform infrared spectrometer, and Raman spectroscopy to determine the synthesis of the composites MWCNT-HA, Cu-H_3_BTC, and MWCNT-HA/Cu-H_3_BTC. The effects of accumulation time, scan rate, pH, and the content of the composites in suspension on the electrochemical detection were investigated. The linear range for fentanyl detection was 0.01–100 μM, and the limit of detection was 3 nM. The quantitative analysis of fentanyl was also achieved in serum, and MWCNT-HA/Cu-H_3_BTC/GCE also showed good stability and reproducibility [77].

**Figure 9:**
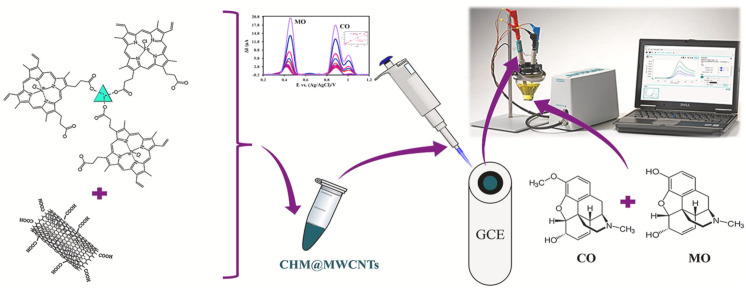
Using Cu-hemin MOF@MWCNTs as an electrochemical sensor for the simultaneous determination of Morphine and Codeine. Reprinted from [79] with permission.

The simultaneous determination of two drugs in the assay will speed up the efficiency of the assay. Karimi et al. synthesized Cu-BTC MOF by a facile hydrothermal synthesis method and dispersed 0.10 g them and 0.90 g graphite powder by ethanol solvent, then mixed with appropriate paraffin oil at 70:30 *w*/*w*% to prepare a carbon paste electrode (CPE). CV and DPV electrochemical methods were used to record the test data when methadone and methaminophen were present together, with the detection limits of 0.05 μM and 0.02 μM, respectively, and they all had two linear ranges, methadone with both 0.08–80 μM and 80–800 μM and methaminophen with both 0.09–100 μM and 100–900 μM. Finally, successful experiments were conducted in blood and urine, showing that Cu-MOF/CPE can successfully analyze both drugs in real samples [78]. Mousaabadi et al. prepared Cu-Hemin MOF and compounded it with MWCNTs to synthesize CHM@MWCNTs composites. After this, they successfully prepared new sandwich CHM@MWCNTs composites by various spectroscopic and microscopic characterization methods for the surface modification of GCE. They investigated the effects of different mass ratios of CHM and MWCTs, pH of the detection environment, and scanning rate on the detection results during the synthesis process. CHM@MWCNTs/GCE achieved the simultaneous determination of Morphine (MO) and Codeine (CO), with the linear range of MO and CO detection being 0.09–30 μM with the detection limits of 9.2 nM and 11.2 nM, respectively, under the optimized conditions. The simultaneous detection of the two opioids was also achieved in human urine and MO injection with the addition of CO, which verified its applicability in real samples, as in Figure 9. [79].

## 5. Conclusions

In this review, we discussed the use of MOFs, MOFs complexes, and surface modified electrochemical sensors based on such materials for the detection of opioids. Electrochemical sensors are notable for their speed, portability, and low cost in comparison to other methods of detection. Different electrodes need to be selected for different detection situations. When the modified electrode is SPCE it can be used as a disposable sensor to avoid the problem of cross-contamination, GCE cannot be used as a disposable electrode. Instead, it can be reused by polishing and is relatively stable and reproducible. In addition, the performance of electrodes modified with MOFs and MOFs complexes has been significantly improved in comparison to that of bare electrodes. These improvements include a wider linear range, lower detection limits, improved stability, and reproducibility to varying degrees. Pure MOFs are more structurally stable than MOFs composites, while MOFs composites have a better limit of detection for opioids concentrations than pure MOFs, making them more suitable for opioids with lower detection lines. There are currently more studies being conducted on electrochemical sensors in opioids, but there are fewer studies being conducted on MOFs and MOFs complexes modified electrodes for this application. Furthermore, most of the current studies are focused on the optimization of the material preparation and the conditions of electrochemical detection. However, very few studies have been conducted on the homogeneity and robustness of MOFs and their complexes in the modified electrodes, even though these characteristics have a significant impact on the sensitivity, stability, and reproducibility of the modified electrodes. Additionally, microfluidics is increasingly used in electrochemical sensors owing to its advantages of miniaturization, integration, and high throughput, while there are still vacancies in its application in opioid detection. In the practical detection of opioids, patient fluids are not easy to obtain repeatedly, and the volume is small, requiring a high level of performance in the detection of micro-samples. Furthermore, the metabolism of opioids in the body changes in real time, so immediate detection of blood levels is required. In addition, because of the specificity of opioids, plasma concentrations are not always measured in the laboratory, necessitating the use of highly integrated and portable devices. Because of the growing need for rapid and accurate detection of opioid concentrations, as well as the great capabilities that have been proven by MOFs and MOFs complexes on electrochemical sensors, this will become a focal point of research soon.

## Figures and Tables

**Table 1:** Classification of MOFs.

No.	Classification Method	Common Material
1	Skeleton space composition	One-dimensional (1D) [24,25], Two-dimensional (2D) [26], Three-dimensional (3D) [27]
2	Ligand	Isoreticular metal-organic frameworks (IRMOFs) [28], Zeolitic imidazolate frameworks (ZIFs) [29], Materials of institut lavoisier (MIL) [30], Porous coordination network (PCN) [31]
3	Organic bridging ligands	Nitrogen-containing heterocyclic [32], Carboxyl-containing [33], Nitrogen-containing heterocyclic and carboxyl-containing [34]
4	Composition of materials	Metal-organic framework (MOF-n), Rare-earth polymeric framework (RPF-n) [35], Metal peptide framework (MPF-n) [36]

**Table 2:** Application of MOFs modified electrodes in the detection of opioid drugs.

Analyte	Method	Electrode	LOD (μM)	Linear Range (μM)	Real Samples	Ref.
Buprenorphine	CV DPV	Zn/La^3^+/MOF/MIP/GCE	0.0021	0.0079–0.0992	Blood	[75]
Fentanyl	DPV	Zn (II)-MOF/SPCE	0.3	1–100	Blood plasma and urine	[76]
Fentanyl	DPV	MWCNT-HA/Cu-H_3_BTC/GCE	0.003	0.01–100	Blood serum	[77]
Methadone Methocarbamol	CV DPV	Cu-MOF/CPE	0.05 0.02	0.08–80; 80–800 0.09–100; 100–900	Blood and urine	[78]
Morphine Codeine	DPV	Cu-hemin MOF@MWCNTs/GCE	0.0092 0.0112	0.09–30	Urine and injection	[79]

## Data Availability

Not applicable.
